# Long Non-Coding RNAs as New Master Regulators of Resistance to Systemic Treatments in Breast Cancer

**DOI:** 10.3390/ijms19092711

**Published:** 2018-09-11

**Authors:** Alma D. Campos-Parra, Eduardo López-Urrutia, Luz Tonantzin Orozco Moreno, César López-Camarillo, Thuluz Meza-Menchaca, Gabriela Figueroa González, Lilia P. Bustamante Montes, Carlos Pérez-Plasencia

**Affiliations:** 1 Laboratorio de Genómica, Instituto Nacional de Cancerología (INCan), Av. San Fernando 22, Col. Sección XVI, Tlalpan, C.P. 14080 Ciudad de México, Mexico; dra.tonantzin@outlook.es (L.T.O.M.); gabufg@gmail.com (G.F.G.); carlos.pplas@gmail.com (C.P.-P.); 2 Unidad de Biomedicina, FES-IZTACALA, Universidad Nacional Autónoma de México (UNAM), Av. De Los Barrios 1. Col. Los Reyes Iztacala, C.P. 54090 Tlalnepantla, Mexico; e_urrutia@me.com; 3 Posgrado en Ciencias Genómicas, Universidad Autónoma de la Ciudad de México, San Lorenzo 290, Del Valle Sur, Benito Juárez, C.P. 03100 Ciudad de México, Mexico; genomicas@yahoo.com.mx; 4 Laboratorio de Genómica Humana, Facultad de Medicina, Universidad Veracruzana (UV), Médicos, Unidad del Bosque, Xalapa, C.P. 91010 Veracruz, Mexico; thuluz@gmail.com; 5 Decanato. Ciencias de la salud. Universidad Autónoma de Guadalajara. Av. Patria 1201, Col. Lomas del Valle, C.P. 45129 Zapopan, Mexico; patricia.bustamante@edu.uag.mx

**Keywords:** breast cancer, lncRNAs, systemic treatments

## Abstract

Predicting response to systemic treatments in breast cancer (BC) patients is an urgent, yet still unattained health aim. Easily detectable molecules such as long non-coding RNAs (lncRNAs) are the ideal biomarkers when they act as master regulators of many resistance mechanisms, or of mechanisms that are common to more than one treatment. These kinds of markers are pivotal in quasi-personalized treatment selection, and consequently, in improvement of outcome prediction. In order to provide a better approach to understanding development of disease and resistance to treatments, we reviewed current literature searching for lncRNA-associated systemic BC treatments including endocrine therapies, aromatase inhibitors, selective estrogen receptor modulators (SERMs), trastuzumab, paclitaxel, docetaxel, 5-fluorouracil (5-FU), anthracyclines, and cisplatin. We found that the engagement of lncRNAs in resistance is well described, and that lncRNAs such as urotelial carcinoma-associated 1 (UCA1) and regulator of reprogramming (ROR) are indeed involved in multiple resistance mechanisms, which offers tantalizing perspectives for wide usage of lncRNAs as treatment resistance biomarkers. Thus, we propose this work as the foundation for a wide landscape of functions and mechanisms that link more lncRNAs to resistance to current and new treatments in years of research to come.

## 1. Background

### 1.1. Breast Cancer

Breast cancer (BC) is the most common cancer diagnosed in women with about one million estimated new cases every year; it is the leading cause of cancer deaths among females. Thus, this disease is an important health problem worldwide [1]. Being such a highly heterogeneous disease in terms of clinical course and biological behavior, BC was only successfully classified through molecular and genomic tools, as luminal A, luminal B, human epidermal growth factor receptor 2 (HER2), basal, and normal-like subtypes [2]. Luminal A tumors have positive estrogen and progesterone receptor expression, negative HER2 expression, and low Ki-67 expression. Luminal B tumors have positive estrogen and progesterone receptor expression, negative or positive HER2 expression, and high Ki-67 expression. Both luminal tumors are the most common subtypes, being luminal A predominant, and in general, both subtypes show a good prognosis. The HER2 subtype includes tumors with negative estrogen and progesterone receptor expression, HER2 positive expression, and high Ki-67 expression; these are tumors with poor prognosis. Basal subtype (triple negative) refers to tumors with negative estrogen and progesterone receptor expression, negative HER2 expression, and positive basal marker expression; this subtype presents with an aggressive clinical course. The normal-like subtype encompasses tumors with positive estrogen and progesterone receptor expression, and negative HER2 and Ki-67 expression; it shows intermediate prognosis. Each subtype has different treatment and responses, as we discuss later [2].

Systemic treatment of BC patients is assigned according to the molecular classification and includes endocrine treatment, targeted therapy, and chemotherapy. In spite of advances in different treatment options, a substantial number of patients exhibit recurrence of disease and decreased survival as consequence of de novo or acquired resistance to treatments, which increases metastasis rates [3]. Once the metastasis is detected, the five-year overall survival (OS) rate is estimated to be below 25% [4]. In this scenario, it is crucial to identify novel molecular biomarkers that characterize or predict therapy response in order to extend patient OS and to avoid or delay the disease recurrence that promotes metastatic BC to be a chronic disease.

### 1.2. Long Non-Coding RNAs

The Encyclopedia of DNA elements (ENCODE) project exposed that more than 80% of the human genome is transcribed into non-coding RNAs with a biochemical function; they are classified as follows: transfer RNA (tRNA), ribosomal RNA (rRNA), small nucleolar RNA (snoRNA, which functions as part of ribo-protein complexes which are exported into the nucleolus to participate in rRNA processing, and tRNA and messenger RNA (mRNA) editing), small nuclear RNA (snRNA, which is involved in alternative splicing of pre-mRNA), microRNAs (miRNAs), and long non-coding RNAs (lncRNAs) [5]. In addition to basic research, lncRNA clinical applications are an area of emerging interest, due to their ideal qualities as diagnostic biomarkers and therapeutic targets [6]. Their differential expression forms patterns that are proven specific for various complex diseases, such as cardiovascular, autoimmune, rheumatoid arthritis, type 1 diabetes, systemic lupus erythematosus, psoriasis, neurodegenerative disorders, Alzheimer’s, Parkinson’s, Huntington’s, psychiatric, schizophrenia diseases, depression/anxiety disorders, and cancer; once the effects of their regulation are elucidated, they are tantalizing therapeutic targets [7].

Long non-coding RNAs (lncRNAs) are a class of non-coding RNA with a length of over 200 bases and quite a complicated biogenesis. Most lncRNAs are transcribed by RNA polymerase II, although some lncRNAs are transcribed by RNA polymerase III; the majority of them are spliced and polyadenylated. They can be classified into five categories according to their genomic location: sense lncRNA, antisense lncRNA, bidirectional lncRNA, intronic lncRNA, and intergenic lncRNA [8]. Expression of several lncRNAs might affect cancer prognosis, as they were proposed as master regulators of cancer pathways through the modulation of gene expression [9]. Gene regulation by lncRNAs can be exerted at different levels including chromatin modification, transcription, posttranscriptional processing, scaffolding or decoy function for mRNAs, and post-transcriptional messenger RNA regulation [10]. Despite lncRNA research being comparatively young, early releases of the GENCODE project consortium based on manual curation, computational analysis, and targeted experimental validation released in April 2018, include 15,779 lncRNA genes for 28,468 lncRNA transcripts (current human version, Genecode28) [11]. Notwithstanding constant advances, the precise molecular mechanisms and functions of lncRNAs remain unclear. The role of lncRNAs as major regulators of drug resistance is being elucidated; in this respect, Leucci E recently summarized the lncRNAs that can be used to sensitize cancer cells to several treatments in different types of cancer [6]. For instance, lncRNA Activated in renal cell carcinoma with Sunitinib Resistance (lncARSR) in renal cancer, nuclear paraspeckle assembly transcript 1 (NEAT1) in breast and ovarian cancer, long intergenic ncRNA for Kinase activation (LINK-A) in triple negative BC, Epithelial Growth Factor Receptor AntiSense 1 (EGFR-AS1) in squamous cell carcinoma, and Prostate Cancer Associated Intregenic noncoding RNA transcripts (PCAT8) and Prostate Cancer Gene Expression Marker 1 (PCGEM1) in prostate cancer [6]. We henceforth focus on lncRNAs that predicted response to systemic treatments in BC.

### 1.3. Long Non-Coding RNAs and Breast Cancer

**Figure 1:**
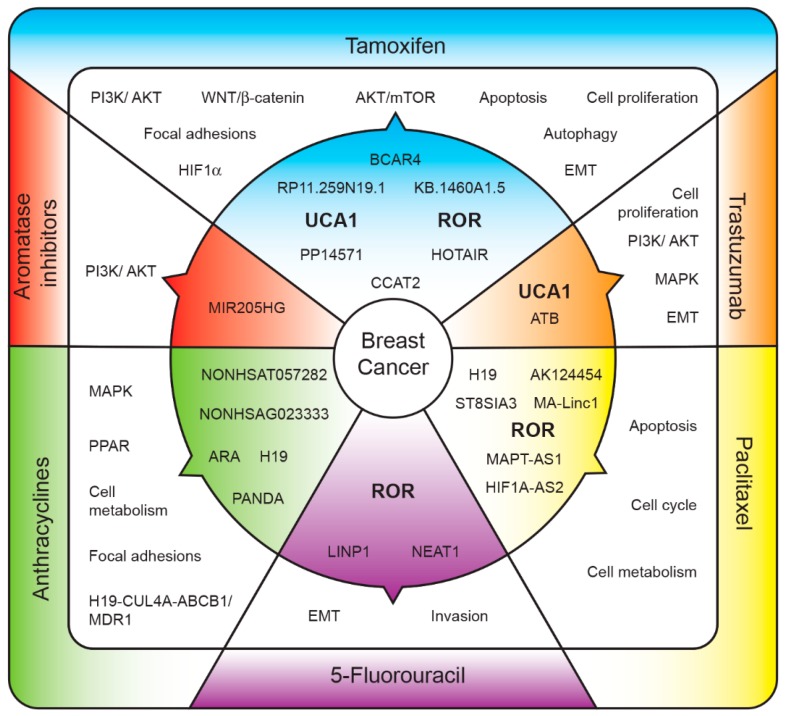
Long non-coding RNAs (lncRNAs) associated with treatment resistance. Upregulated lncRNAs (inner section) regulate several pathways (middle section) that ultimately lead to resistance to systemic treatments (outer section). LncRNAs: BC anti-estrogen resistance 4 (BCAR4), Urotelial carcinoma-associated 1 (UCA1), RNA regulator of reprogramming (ROR), Hox antisense intergenic RNA (HOTAIR), Colon cancer-associated transcript 2 (CCAT2), lncRNA activated by transformig growth factor-beta (ATB), Mitosis-Associated Long Intergenic Non-Coding RNA 1 (MA-Linc1), MAPT antisense RNA 1 (MAPT-AS1), HIF antisense RNA 2 (HIF1A-AS2), lncRNA in non-homologous end joining (NHEJ) pathway 1 (LINP1), ncRNA nuclear paraspeckle assembly transcript 1 (NEAT1), adriamycin resistance-related lncRNA (ARA), lncRNA P21-associated lncRNA DNA damage activated (PANDA). Pathways: Phosphatidylinositol 3-kinase/Serine/Threonine Kinase 1 (PI3K/AKT), Mitogen-activated protein kinase (MAPK), peroxisome proliferator-activated receptor (PPAR), epithelial-mesenchymal transition (EMT), Treonine Kinase1/mammalian target of rapamycin complex 1 (AKT/MTOR), Hypoxia-inducible factor 1-alpha (HIF1α), Cullin-4A (CUL4A), ATP Binding Cassette Subfamily B Member 1 (ABCB1), Multi-Drug Resistance Gene (MDR1).

A number of papers report genome-wide sequencing or microarray analysis that describes the differential expression of lncRNAs in breast tumor tissue vs. normal breast tissues [12,13,14], even between subtypes [15,16]. Moreover, lncRNA expression levels are related to development, prognosis, metastasis, and recurrence [13]. Wang et al. summarize the principal dysregulated lncRNAs with oncogenic function such as: *Hox* antisense intergenic RNA (HOTAIR), *H19*, urotelial carcinoma-associated 1 (UCA1), colon cancer-associated transcript 2 (CCAT2), adriamycin resistance-related (ARA), and regulator of reprogramming (ROR); and those with tumor suppressor function: metastasis associated lung adenocarcinoma transcript 1 (MALAT1), maternally expressed gene 3 (MEG3), growth arrest-specific transcript 5 (GAS5), and NF-KappaB interacting lncRNA (NKILA), among others [17]. The function of numerous lncRNAs was analyzed by in vitro cell-based assays, mouse models, and cancer sample expression surveying. This review summarizes the current knowledge and research approaches to lncRNAs as potential response biomarkers to systemic treatments in BC (Table 1 and Figure 1), based on our search for experimental research papers indexed in the Medline database. We also highlight their mechanisms delineating their potential target molecules and consequently affected cancer signaling pathways. The overall vision is to understand the heterogeneous and complex disease in order to optimize therapeutic regimens for personalized treatment leading to opportunely prevent BC recurrence.

## 2. Main Text

### 2.1. Endocrine Therapy

Breast cancer with expression of estrogen receptor (ER^+^) is diagnosed in around 70% of BC patients; this cancer subtype depends on the hormone estrogen for growth and proliferation [42]. There are two principal estrogen receptor isoforms—ERα and ERβ—that drive nuclear and extranuclear pathways. Nuclear pathways comprise the interaction of ligand-bound ER dimers with estrogen-responsive elements in target gene promoters, and extracellular pathways consist of the activation of kinase cascades mediated by the translocations of nuclear receptors to the cytoplasmic side of the cell membrane [43].

There are three treatment options for these patients: (a) aromatase inhibitors, (b) selective estrogen receptor modulators (SERMs), and (c) selective estrogen receptor degraders (SERDs) that antagonize ER [44]. Unfortunately, over 30% of ER^+^ tumors are intrinsically hormone-resistant at diagnosis and around 40% of the tumors that initially respond to hormone therapies eventually present with resistance [45,46].

### 2.2. Aromatase Inhibitors

Aromatase is an enzyme (a cytochrome P450, heme-containing protein) that, through the aromatization of the A-ring of androgens, catalyzes the biosynthesis of estrogens—estradiol and estrone—from the androgenic precursors—testosterone and androstenedione. Third-generation aromatase inhibitors (AIs), which exhibit great effectiveness and specificity, along with lower toxicity, comprise anastrozole, letrozole, and exemestane [47]. These AIs can be divided into two categories: steroidal AIs (such as exemestane) and nonsteroidal AIs (such as anastrozole and letrozole). There are three United States (US) Food and Drug Administration (FDA)-approved oral AIs in clinical use for the treatment of post-menopausal women with hormone receptor-positive BC, and clinical trials reported that AIs are usually more tolerable and more effective in terms of clinical response rate (RR) and median time to progression than tamoxifen [48,49]. For instance, AIs were shown to reduce the recurrence by 30% vs. five years of tamoxifen alone. However, intrinsic and acquired resistance to AIs is a major clinical obstacle, and to date, no biomarkers are used clinically to guide treatment in these patients [50,51].

At the time of writing, only one report described an lncRNA whose expression is correlated with aberrant signaling of cancer cells that induce AI treatment resistance [18]. Briefly, in a cohort of 4658 BC patients, two single-nucleotide polymorphisms (SNPs) were identified (rs4476990 and rs3802201) in the gene *MIR205HG*, which is an lncRNA. The expression of this polymorphic gene may alter ERα expression, which consequently impacts response to AIs (anastrozol or exemestane) as adjuvant therapy for early-stage BC. In vitro analysis demonstrated that *MIR205HG* is a functional polymorphic gene whose overexpression increased ERα expression leading to augmented cell proliferation in ERα^+^ BC cells; however, when BC cells were treated with AI therapy (anastrozol or exemestane), a decrease in both *MIR205HG* lncRNA and *ESR1*—the gene encoding ERα—expression was evident. Overexpression of this lncRNA improved cell proliferation, colony formation, and ERα expression, corroborating the positive *MIR205HG*–ERα association. Moreover, in order to understand the mechanism of action of this association, the authors evaluated phosphorylated (p)-AKT and p-FOXO3 levels in *MIR205HG* knocked-down cell lines, and observed an increase in p-AKT levels (on both Ser473 and Thr308 residues) which promoted downregulation of p-FOXO3 (on Ser318/Ser321) and total FOXO3; in consequence, the *ESR1* expression was downregulated. Then, the authors proposed that *MIR205HG* regulates ERα expression through different levels that involve the AKT pathway. This way, the SNPs related to *MIR205HG* were rendered as potential AI response biomarkers [18]. The lack of studies to identify lncRNAs associated with response to this treatment is evident; more studies are needed to have a comparison point for *MIR205HG*.

### 2.3. Selective Estrogen Receptor Modulator (SERM) Therapy

#### Tamoxifen

Selective estrogen receptor modulators (SERMs) are synthetic molecules that bind ERα and ERβ to regulate its transcriptional potential in several ways in estrogen target tissues [52]. The first clinically used SERM was tamoxifen; this drug is approved by the FDA as a first-line endocrine treatment in pre- and post-menopausal luminal BC patients [53]. Although this drug reduces the risk of recurrence in around 41% and mortality in 34%, about 30–40% of patients who take tamoxifen become resistant to endocrine therapy within 3–5 years, which leads to cancer recurrence or metastasis with poor disease-free or OS [45,54].

The most studied mechanisms of tamoxifen resistance are loss of ER expression, ER mutations, and cross-talk between ER and growth-factor signaling pathways [54,55]. Some biomarkers were proposed to predict the drug resistance of these ER-positive patients primarily treated with tamoxifen, such as ER expression, progesterone receptor (PR) expression, and HER2 and multigene signatures [56]. The role of lncRNAs as biomarkers of response to treatments in BC is gaining a lot attention. In this context, Wang K. and colleagues recently described a signature based in 11 lncRNAs for prediction of relapse risk, through raw microarray of GSEI7705 recovered from cohorts of the Gene Expression Omnibus (GEO) database and data from the Cancer Genome Atlas (TCGA). The high expressions of RP11.259N19.1, KB.1460A1.5, and PP14571, and low expressions of PINK1.AS, KLF3.AS1, LINC00339, LINC00472, RP11.351I21.11, PKD1P6.NPIPP1, PDCD4.AS1, KLF3.AS1, PP14571, and RP11.69E11.4 predict disease relapse to tamoxifen in ER^+^ BC patients [19]. Through gene set enrichment analysis (GSEA) based on GSE17705, the signaling pathways related to these lncRNAs were identified and visualized in Cytoscape (version 2.8.2); the PI3K/AKT, focal adhesions, and WNT (The name Wnt is resultant from a fusion of the name of the Drosophila segment polarity gene wingless and the name of the vertebrate homolog, integrated or int-1) signaling pathways that stood out (*p* < 0.05). With these results, the authors reported a signature to predict the benefits of tamoxifen treatment based on 11 lncRNAs [19].

Urotelial carcinoma-associated 1 (UCA1) is involved in tamoxifen resistance; its aberrant expression is associated with drug resistance in several types of cancer such as bladder cancer [57], gastric cancer [58], colorectal cancer [59], and BC [20]. Long non-coding RNA UCA1 is encoded in chromosome 19p13.12, and regulates several functions such as proliferation, apoptosis, invasion, cell cycle, and drug resistance [60,61]. In 54 samples of stage I–IV BC, UCA1 expression was evaluated by RT-PCR, and it was demonstrated that its expression was higher in advanced stages (III–IV). In the same samples, using an immunohistochemistry assay, higher β-catenin expression in the nuclei was also observed. This finding was confirmed using tumor tissues from xenograft mouse model, and higher β-catenin nuclear expression was observed by Western blot when UCA1 was overregulated. Furthermore, in vitro analysis using a UCA1 knockdown displayed diminished cell survival and migration ability, and promoted apoptosis of tamoxifen-resistant BC cells. These findings indicated that UCA1 expression contributed to tamoxifen resistance through the stimulation of the WNT/β-catenin signaling pathway, allowing the extracellular redistribution of ER; thus, the inhibition of the WNT pathway or UCA1 expression could overcome the resistance to tamoxifen [20].

The AKT/mTOR cell signaling pathway is also involved in UCA1-activated tamoxifen resistance. This finding was examined through Western blot detection of p-AKT and m-TOR in LCC2, LCC9 (tamoxifen-resistant BC cells), and MCF-7 (tamoxifen-sensitive BC cell line). The authors observed concurrent UCA1 overexpression and significantly higher expression of p-AKT and p-mTOR in LCC2 and LCC9 cells. Conversely, UCA1 silencing significantly reduced the p-AKT and p-mTOR expression in LCC2 and LCC9 cells. These findings suggested that UCA1 regulates the AKT/mTOR signaling pathway positively, leading to increased tamoxifen resistance [21].

When searching for the mechanism behind UCA1 overexpression in ER^+^ breast-cancer-derived cell lines, was found a very interesting regulation loop: UCA1 sponges miR-18, effectively blocking its availability, and thus, decreasing its effects on its target mRNAs; HIF1A is among these targets and regulates the transcription of UCA1. In addition to describing the association of an lncRNA with tamoxifen resistance, this work offers a glimpse into a complex network of interactions that regulates gene expression [22].

Exosomes have an important role in lncRNA, miRNA, and RNA transfer, taking an important function in the mechanism of acquired drug resistance. In BC, MCF-7 BC cells pre-treated in vitro with exosomes from tamoxifen-resistant LCC2 cells showed increased tamoxifen resistance. Furthermore, UCA1 expression was detected in exosomes released from tamoxifen-resistant BC cells, demonstrating how exosomes spread tamoxifen resistance [62].

Colon cancer-associated transcript 2 (CCAT2)—An lncRNA that was identified for the first time in colorectal cancer—is increasingly detected in human cancers, and its overexpression is associated with poor clinical outcome. Tamoxifen-resistant BC cells (MCF-7 and T47D) were observed to overexpress CCAT2, while CCAT2 knockdown promoted apoptosis and diminished cell proliferation, improving the tamoxifen sensitivity [23].

Higher expression of a large intergenic non-coding RNA regulator of reprogramming (lncRNA-ROR) was detected in 74 BC tissue vs. adjacent tissue samples (4.96 vs. 1.02; *p* < 0.01) [24]. In vitro analysis detected a peculiar function of this lncRNA; it acts as a sponge which blocks miR-205-5p expression. Subsequently, it increases the expression of target genes such as *ZEB1* and *ZEB2* in order to potentiate the epithelial–mesenchymal transition (EMT) process, which is increased in tamoxifen-resistant BC cells. Preclinical studies demonstrated that inhibition of ROR expression increased LC3 and Beclin expression, inducing autophagy, suppressing cell invasion and migration, and reducing tumor size, thus reverting tamoxifen resistance [24]. A similar study demonstrated ROR overexpression in BC tissue and BC cell lines (MCF-7, BT-20, MDA-231, and BT474), and also demonstrated that blocking ROR suppressed cell proliferation, invasion, and migration, and reverted tamoxifen resistance via an autophagy mechanism in tamoxifen-resistant BC cells (BT474) [63].

*Hox* antisense intergenic RNA (HOTAIR) is the most studied lncRNA, due to it being the first identified lncRNA involved in cancer epigenetic regulation [64]. It is a 2.2-kb gene located in chromosome 12, and its principal function is chromatin remodeling; its 5′ region interacts with polycomb repressive complex 2 (PRC2), while the 3′ region has affinity for the lysine-specific demethylase/repressor element-1 silencing transcription factor (LSD1)/so-REST/REST) complex to coordinate histone H3 lysine-27 methylation and lysine-4 demethylation, thereby controlling transcription through chromatin structure alterations and promoting cancer metastasis [65,66].

Gupta et al. reported HOTAIR as the most upregulated lncRNA in stage I–II BC as per their RT-PCR analyses, and its expression was already associated with metastasis (*p* = 0.0004) [65]. Also, Sørensen KP. et al. reported HOTAIR as a strong predictor of poor prognosis (*p* = 0.012) correlated with ER expression (*p* = 0.0086), suggesting that HOTAIR is a potential predictor for metastasis in ER^+^ BC patients [67]. Nonetheless, Gökmen-Polar Y et al., through tissue microarray from 133 BC patients, revealed that HOTAIR had a role as a biomarker of lymphatic metastases in ER-negative patients (*p* = 0.018) vs. ER-positive patients (*p* = 0.018); these data were validated using TCGA data from BC subjects [68].

HOTAIR expression is regulated in BC through ER-interceded transcriptional repression, whereby it is restored upon the blockade of ER signaling via FOXA1 or FOXM1; hence, HOTAIR overexpression is notable in tamoxifen-resistant BC. Through RNA pulldown and RNA immunoprecipitation assays, it was demonstrated that this lncRNA interacts with the ER protein in order to potentiate ER transcriptional action in absence of estrogen, evidencing that HOTAIR has a crucial role in the development of tamoxifen resistance [25]. Furthermore, its expression strikes us as a promissory and advantageous biomarker or therapeutic target.

The BC anti-estrogen resistance 4 (*BCAR4*) gene expresses a protein but, interestingly, also an lncRNA of the same name [69]. It was observed at high expression levels in patients treated with tamoxifen that manifested poor prognosis [26]. Another study performed a knockdown *BCAR4* which was able to inhibit cell proliferation and tamoxifen resistance, upon exposure to lapatinib [26].

Many more lncRNAs are associated with resistance to tamoxifen than with resistance to other drugs; moreover, its biological role is more deeply described. This may be due to various circumstances such as drug availability, cost benefit, or the sheer fact that tamoxifen is the first-line endocrine treatment in pre- and post-menopausal luminal BC patients.

### 2.4. Long Non-Coding RNA-Targeted Therapies

#### Trastuzumab

Trastuzumab is a monoclonal antibody against ERBB2 (HER2 or HER-2/neu), approved as target therapy for HER2 protein expression and *HER2* gene amplification in BC patients [70]. In a similar fashion to other EGF-like family members, the ERBB2 protein phosphorylates and activates a wide range of cellular processes and pathways such as metastasis, cell cycle, survival, proliferation, angiogenesis, and apoptosis. Trastuzumab targets this receptor, limiting its ability to start many of these pathways, or via antibody-mediated cancer cell lysis through natural killer cells [71,72].

HER2-positive BC patients represent around 15–20% of all cases [73]. Although the expression of HER2 is correlated with more aggressive tumors, trastuzumab therapy can result in improvement, although its adverse effects include cardiac toxicity, in around 30% of patients, according to a 2011 study [74]. Trastuzumab alone has a response rate of about 26%, a rate that goes up to 82% in combination with drugs such as lapatinib. Mechanisms of trastuzumab resistance are present in rates as high as 60%, which means that a substantial number of patients will eventually relapse or develop resistance [75,76,77]. Hence, it is essential to gain better understanding of these resistance mechanisms toward truly personalized treatment for these patients.

In this regard, the role of the lncRNAs is far from completely elucidated. A recent work; it was described by microarray assay using trastuzumab-resistant BC cell lines and trastuzumab-resistant BC tissues. Among 30 lncRNAs detected in 50 BC and 50 normal tissue samples, the most upregulated lncRNA associated with trastuzumab resistance was lncRNA activated by transformig growth factor-beta (TGFB) (lncRNA-ATB). Functional analysis determined that lncRNA-ATB sponged miR-200c (a microRNA generally overexpressed in trastuzumab-resistant cells) in order to favor upregulation of *ZEB1* and ZNF-217, to induce EMT so as to promote trastuzumab resistance and metastasis. This way, this lncRNA is a likely responsible for malignant phenotype development [27].

A similar report, also by microarrays of trastuzumab-resistant cell lines and trastuzumab-resistant BC tissues, showed that the lncRNA-growth arrest-specific transcript 5 (GAS5) was downregulated. This downregulation was associated with advanced stage, histological grade, and poor disease-free survival and OS. GAS5 knockdown promoted tumorigenesis and metastatic potential in SK-BR-3 BC cells. Likewise, athymic mice inoculated with GAS5-silenced cells resulted in an increase in tumoral mass. Moreover, low GAS5 levels correlated with low Phosphatase and tensin homolog (PTEN) levels in BC tissues. In vitro assays were performed to understand this biological mechanism, and it was demonstrated that GAS5 sponged miR-21 (which targets PTEN), favoring PTEN expression and suppressing cell proliferation. GAS5 has great potential as a prognostic marker [28].

A later report, performed with very sophisticated next-generation RNA-sequencing, surveyed RNA from sensitive and trastuzumab-resistant HER2^+^ cell lines and biopsy tumors, and identified an lncRNA and mRNA profile strongly associated with trastuzumab resistance. The authors focused on S100P mRNA, which was upregulated by epigenetic changes at enhancers. Functional analysis showed that its inhibition reverted trastuzumab resistance, and interestingly, this gene was able to activate the RAS/MEK/MAPK pathway as a compensatory mechanism of HER2 inhibition by trastuzumab [78]. So far, the role of lncRNAs in trastuzumab resistantance is still to be elucidated, and further studies are needed in order to understand their potential utility.

The UCA1 lncRNA seems to have a role in trastuzumab resistance as well. It was silenced by siRNA in the trastuzumab-resistant BC cell line SK-BR-3, and consequent;y, trastuzumab-triggered apoptosis was increased and miR-18a expression was upregulated. This suggested that, in trastuzumab-resistant cells, UCA1 is upregulated and sponges miR-18a, which targets YAP1—a key protein associated with upregulation of PI3K and MAPK signaling—allowing its overexpression. Then, the role of the UCA1/miR-18a/YAP1 axis would be needed to validate in vivo HER2^+^ BC models [29].

Summarizing research on trastuzumab-resistance-promoting lncRNAs was feeble as well; we found reports about three involved lncRNAs—UCA1, ATB, and GAS5—and their biological mechanisms described above.

### 2.5. Taxanes for Breast Cancer

#### 2.5.1. Paclitaxel

Paclitaxel (taxol) is a taxane-class agent that stabilizes microtubules, preventing mitosis (M)-phase entry and eventually leading to cell death; as an anti-neoplasic, it is a prescribed for several cancer types such as lung, breast, ovarian, and liver cancer [79]. It is indicated as a first-line therapy for many cancers including breast cancer. An overall response rate of between 30% and 42% was reported for single-agent docetaxel [80].

However, its effectiveness is still hindered by resistance, and a number of lncRNAs were shown to participate in several resistance mechanisms. For instance, the expression level of lncRNA H19 was previously reported in various cancers, where it is thought to take part in tumorigenesis and metastasis [81]. Moreover, H19 expression correlates with paclitaxel resistance in BC. A recent study evaluated this correlation and found that H19 regulates the expression of the proapoptotic genes *LIK* and *LOXA* negatively, thus mediating the previously known ERβ-dependent resistance [30]. Also, H19 is upregulated in doxorubicin-resistant cells and its knockdown re-sensitizes them. According to the elegant model proposed by Zhu et al., H19 upregulates the expression of CUL4A, a ubiquitin ligase component that was observed to be a positive regulator of the multi-drug resistance protein 1 (MDRP1), although the molecular mechanisms are still to be fully understood [41].

Bida and collaborators described the novel lncRNA, Mitosis-Associated Long Intergenic Non-Coding RNA 1(MA-linc1), which participates in cell-cycle regulation favoring M-phase exit. Although the authors did not originally search for a paclitaxel resistance mechanism, they found that paclitaxel-induced apoptosis was enhanced by 90% when MA-linc1 was silenced concomitantly; this suggested that lncRNA leads to mitosis completion through microtubule destabilization, and that the effect of MA-linc1 silencing on paclitaxel-induced apoptosis is also mediated by its effects on its neighboring gene Purα, which is often deleted in cancer [31].

On the other hand, Jiang et al. performed a prospective study involving 275 patients aimed at finding a predictive and prognostic mRNA–lncRNA signature. The signature was finally integrated by FCGR1A, RSAD2, and CHRDL1 mRNAs, and HIF1A-AS2 and AK124454 lncRNAs. Both lncRNAs were found to interfere with paclitaxel-induced gap 2 (G2)–M arrest in in vivo assays, presumably by altering the expression of metabolism and cell-division-related genes, respectively, according to the results of in silico analysis performed by the authors [32].

Significantly upregulated in BC samples, linc-ROR, also known as lincRNA-ST8SIA3 was found to induce an EMT phenotype and expression of the EMT markers vimentin and neural (N)-cadherin to the MDA-MB231 cell line. By means of overexpression and shRNA-mediated knockdown experiments, Chen and collaborators established that linc-ROR both enhances the invasion ability of these cells and decreases their sensibility to paclitaxel [33].

The MAPT antisense RNA 1 lncRNA (MAPT-AS1) is an antisense transcript to the *MAPT* gene, which, in turn, codes for the TAU protein; this protein competes with paclitaxel for microtubule binding, rendering cells resistant to its effects [34]. MAPT-AS1 was found to be overexpressed in relation to paclitaxel resistance in ER-negative BC by Pan and colleagues [82]. They observed that MAPT-AS1 expression correlated with MAPT expression as well, and established that MAPT-AS1 knockdown sensitized cells to paclitaxel. The underlying mechanism turned out to be very interesting: MAPT-AS1 binds the MAPT transcript and stabilizes it, thus contributing to high TAU levels [34].

#### 2.5.2. Docetaxel

Similar in chemical nature to paclitaxel, docetaxel is also a microtubule-stabilizing taxane; however, as shown by several studies, it produces significantly extended survival times and decreased secondary effects in metastatic BC. As a primary neoadjuvant, it improves OS (91% vs. 87% *p* = 0.05) compared to anthracyclines [82,83].

Although taxanes are a first-line treatment in metastatic, triple-negative, or HR^+^HER2^−^ BC, resistance to them is not as studied as resistance to tamoxifen. This shows that research in this area is a wide open area of opportunity at the time of writing.

### 2.6. Other Agents

#### 2.6.1. 5-Fluorouracil and Capecitabine

The fluoropyrimidine, 5-fluorouracil (5-FU), is an antimetabolite drug, widely used for BC patients in clinical settings [84]. The compound 5-FU is an analog of uracil with a fluorine atom at the C-5 position, and its mechanism of action involves the inhibition of thymidylate synthase and the incorporation of its metabolites into RNA and DNA, thus inhibiting their normal function [84]. Capecitabine is an oral fluoropyramidine that mimics continuous 5-FU infusion, and phase II studies demonstrated a 20–30% (95% confidence intervals (CIs): 19–43%) response rate as a first line of treatment for BC [85], similar to the 5-FU response rate (32%) in BC patients [86,87]. The difference between both drugs is that capecitabine is not cytotoxic. Although poly-chemotherapy regimens based on these drugs increased the OS of BC patients, most of them experience recurrence [88].

There are several reports focused on analyzing the up- and downregulation of lncRNAs related to 5-FU chemoresistance in colorectal cancer [89] and pancreatic cancer [90]; nevertheless, few reports explore the expression of lncRNAs in 5-FU-treated BC. Among the recently described is the ncRNA nuclear paraspeckle assembly transcript 1 (NEAT1) which was upregulated in BC cells and BC patients, preferentially in stage III–IV tumors vs. stage I–II tumors. This overexpression was associated with poor prognosis and metastasis. In order to comprehend its biological function, through in vitro assays, Li and colleagues demonstrated that NEAT1 induces 5-FU resistance by sponging miR-211; this is a tumor suppressor miRNA that targets high mobility group A (HMGA), a positive regulator of EMT, present in 5-FU-resistant BC cells [35]. Then, NEAT1 lncRNA may represent an unfavorable marker of BC.

The lncRNA in non-homologous end joining (NHEJ) pathway 1 (LINP1), was reported as a promoter of BC progression and chemoresistance through negative regulation of apoptosis-related proteins. In addition, it stimulates metastasis inducing the expression of EMT-related markers and decreasing the inhibitory effects of p53 on cancer cell metastasis, showing association between p53 and LINP1. It was also found to be highly upregulated in BC patients with distant metastases and advanced clinical stage; moreover, its overexpression was correlated with lower OS and disease-free survival [36].

The third report that we found described that ROR expression was higher in BC tissues, in lymph-node metastasis, and in BC cell lines (MDA-MB-231). The ROR-expressing BC cells displayed resistance to 5-FU, epithelial (E)-cadherin underexpression, and increased vimentin and N-cadherin expression; invasion capability also improved. This makes ROR upregulation an important drug-resistance marker [33,37]. Unfortunately, lncRNA expression in association with capecitabine resistance is yet to be described, as we did not find any report about it at the time of writing. Thus, there is still much to learn about resistance to this and other agents.

#### 2.6.2. Anthracyclines

First known as antibiotics, anthracyclines, including, daunorubicin (DAU), doxorubicin (DOX), epirubicin (EPI), and idarubicin (IDA), are currently an important class of drugs which exhibit a strong efficacy in anticancer chemotherapy, mainly used for BC [91]. Although their mechanisms of action are still controversial, the anticancer activity of anthracyclines may involve the inhibition of macromolecule synthesis through DNA intercalation, free-radical generation, and inhibition of topoisomerase II—causing DNA damage, binding, alkylation, and cross-linking—and induction of apoptosis [92]. Up-to-date literature sustains that patients with intermediate- or high-risk BC, or with high recurrence should be considered for anthracycline-based regimen depending on factors such as age, comorbidities, tumor grade, lymphovascular invasion, and genomic profile. Anthracyclines are not required for all BC patients, and should be eluded in patients with high cardiac risk. Nonetheless, many patients are resistant to these agents, which manifests itself in short recurrence times [93,94].

Several lncRNAs are implicated in BC recurrence. However, the regulatory roles of lncRNAs in chemotherapy resistance of BC to anthracyclines still remain unclear. There are two seminal works where lncRNA expression in adriamycin-resistant BC cells (MCF-7/ADR) was analyzed using microarrays, and both works compare their lncRNA profile with that of parental chemosensitive cells (MCF-7) in order to identify and characterize dysregulated lncRNAs that might be directly involved in BC chemoresistance. Briefly, Jiang and collaborators summarized, for the first time, a global aberrant expression of lncRNAs in cells with acquired adriamycin resistance. Among hundreds of differentially expressed lncRNAs, they identified and explored the role of a new adriamycin resistance-related lncRNA (ARA), suggesting that it may induce resistance by upregulating long-chain fatty-acid CoA ligase 4 (ACSL4), which regulates overall gene expression and multiple signaling routes such as MAPK signaling, focal adhesion, PPAR, and metabolism signaling pathways [38].

On the other hand, more recent microarray analyses allowed identification of another lncRNA as key player in anthracycline-resistant BC. However, most of the dysregulated lncRNAs in current databases are not yet functionally annotated; for this reason, the authors predicted their functions based on their correlated mRNAs and experimental validation. In this work, they showed specific interactions between lncRNAs and genes, as well as lncRNAs and transcription factors. Based on this, it was validated that the lncRNA NONHSAT028712 regulated nearby Cyclin-dependent kinase 2 (CDK2), interfering with cell cycle and chemoresistance. Also, the authors identified another group of lncRNAs—NONHSAT057282 and NONHSAG023333—that interact with chemoresistance-modulating transcription factors such as ELF1 and E2F1 [39].

Some other studies reported functional screenings in which mechanisms of anthracycline resistance can be identified; such is the case of lncRNA *P21*-associated lncRNA DNA damage activated (PANDA). The overexpression of this lncRNA inhibited the expression of apoptotic genes such as *APAF1*, *BKI*, *FAS*, and *LRDD* through competitive binding of the transcription factor NF-YA; there was a direct association between poor outcome in patients with BC and high PANDA overexpression [40]. The most recently described lncRNA is the imprinted oncofetal *H19*, which is overexpressed in 70% of BC patients and its expression was associated with poor prognosis [95]. Zhu Q. et al. reported that H19 was found significantly overexpressed in doxorrubicine-resistant MCF-7 BC cells. Through pharmacological and genetic approaches, it was suggested that *H19* lncRNA mediates chemoresistance through the *H19*/*CUL4A*/*ABCB1*/*MDR1* axis [41]. As mentioned before, lncRNAs associated with anthracycline resistance are still uncertain.

#### 2.6.3. Gemcitabine

Gemcitabine (2′,2′-Difluoro 2′-deoxycytidine, dFdC) is a pyrimidine nucleoside antimetabolite, analog to cytosine arabinoside, originally attributed an antiviral activity; however, it is currently indicated as a single chemotherapeutic agent for patients with pancreatic cancer in the metastatic stage [96], small-cell lung cancer (SCLC) [97], bladder cancer [98], head and neck cancers [99], ovarian cancer [100], and BC [101]. Clinical studies suggest that gemcitabine treatment could be prescribed to recurrent BC patients that were previously treated with taxanes, and these patients manifest an overall RR of 25% [102]. Moreover, combined regimens that contain gemcitabine showed a better significant response rate that gemcitabine alone, although with increased hematologic toxicity [103]. For instance, a phase II study of the gemcitabine-plus-paclitaxel doublet demonstrated consistent high response rates (40% to 71%) and manageable toxicity as a first-line therapy in advanced BC patients. In spite of this, disease recurrence was observed [101]. At the time of writing, we found no reports of lncRNA associated to docetaxel resistance in BC.

#### 2.6.4. Cisplatin

Cisplatin, also known as *cis*-diamminedichloroplatinum(II), was first synthesized in 1844, but it was not until 1978 that the FDA approved platinum compounds for cancer treatment [104]. Cisplatin is a metallic (platinum) coordinate compound with a square planar geometry; it is used for treating a variety of malignancies, including BC [104]. The most well-described modes of action are DNA damage, DNA synthesis and mitosis inhibition, and apoptosis induction [105]. Platinum salts are used as a neoadjuvant and palliative treatment for BC. Carboplatin displayed a 43.7% (95% CIs 38.1–49.4) response rate [106], while cisplatin proved more effective with a 64% (95% conditional CIs, 44–81%) response rate [107]. Both treatments cause cell death through disruption of homologous recombination pathways, a concept known as synthetic lethality [108]. Cisplatin is particularly useful for triple-negative BC (TNBC), a group of tumors with high genomic instability often associated with HR deficiencies, accounting for approximately 15–20% of all BC cases [109]. We found a sole report on lncRNAs associated with NEAT1; Adriaens et al. showed that NEAT1 was able to sensitize MCF7 BC cell lines to different chemotherapeutics and PARP inhibitors through p53 reactivation [110].

## 3. Future Perspectives

Ever since the discovery of lncRNAs, accumulating evidence provided a new horizon for understanding the orchestrated regulation of several genes involved in carcinogenesis and in malignant phenotype maintenance. Nonetheless, in spite of the progresses of lncRNA studies, few reports highlight their role as master regulators of drug resistance; thus, there is still much to say in the realm of drug-resistant lncRNAs.

Here, we performed a literature review searching for current reports that identified, analyzed, and proposed lncRNAs as potential systemic treatment-resistance biomarkers in BC (endocrine therapy, targeted therapies, and chemotherapy). In addition to the actual data discussed above, we found that lncRNA studies published in high-impact journals were not as frequent as those from other seemingly similar research areas, such as miRNAs [111]. This might be a consequence of the lack of widely accepted standardized methodologies, but it might also reflect that lncRNAs are yet to be recognized as important regulators of gene function, meriting solid experiment planning that yields strong results. We hope that this review helps lncRNA research gain traction among the scientific community so that more resources are devoted to it, widening our understanding of their functions.

Our analysis allowed us to identify lncRNAs whose higher expression is associated with resistance to one or more systemic treatments (Table 1 and Figure 1). For instance, UCA1 was reported to enhance resistance to tamoxifen [20,21] and trastuzumab therapies [29]. Higher UCA1 expression also predicts gemcitabine resistance in bladder cancer [57], but there are no reports about its biomarker role in BC. Therefore, lncRNAs could be a very broad field of future research with clinical applications.

It is particularly interesting that lncRNAs participate in multi-level post-transcriptional regulation: they downregulate miRNAs through sponging—which, in turn, regulate mRNAs—effectively upregulating the messengers, i.e., they regulate the regulators. For example, UCA1 regulates several routes such as AKT/mTOR, PI3K, MAPK, and WNT/β-catenin signaling by sponging miR-143 [112] and miR-18 [22]. Also, ROR overexpression has an important role favoring resistance to tamoxifen, paclitaxel, and 5-FU through epithelial–mesenchymal transition [63], and invasion capability regulation [33,37]. Several studies reported the role of HOTAIR as a relevant lncRNA in BC [65,67,68] since it is associated with tamoxifen resistance through interaction with the ER protein that activates transcription in absence of estrogens [25]. Likewise, the role of HOTAIR in cisplatin resistance was reported in ovarian [113] and lung adenocarcinoma [114]. Perhaps it is not studied in BC due to this drug being less used in this cancer.

Taken together, these data show us the complexity that known treatment evasion mechanisms can reach, and highlight the importance of further deepening our understanding of them. Therefore, we propose these lncRNAs as potential biomarkers of resistance to systemic treatment in BC and find it reasonable to speculate that blocking UCA1 or ROR will allow us to sensitize breast tumors to several drugs at the same time.

This review leaves several lines of research open, since several lncRNAs (Table 1) involved in drug resistance are categorized as such from association studies, and their precise functions are still unknown. The fact that they are not yet functionally annotated in current databases complicate the analysis; however, He DX et al. were able to predict the biological function of NONHSAT028712, NONHSAT057282, NONHSAG023333, and NONHSA6023333 using informatics tools [39].

The role of lncRNAs in resistance to common drugs docetaxel, gemcitabine, and cisplatin, and other less described therapies, such as bevacizumab, lapatinib, and everolimus, is urgently needed so as to complete an integrative scenario of lncRNAs as potential biomarkers of resistance to all systemic treatments. Data from our search make it reasonable to speculate that such a role exists, at least for the more common aforementioned therapies, as lncRNAs do mediate resistance to them in other cancer types. For instance, UCA1 [115], POTEF-AS1 [116], and MALAT1 [117] contribute to resistance to docetaxel in prostate cancer cells; UCA1 is also reportedly overexpressed in human bladder carcinoma, where it promotes cancer cell proliferation, migration, invasion, and gemcitabine resistance [57]. Meanwhile, lncRNA-LET is downregulated in chemoresistant urinary bladder cancers, and its overexpression delayed gemcitabine-induced tumor recurrence [118]. The correlation between HOTAIR and cisplatin resistance was described in ovarian cancer [113] and lung adenocarcinoma [114], where it causes inhibition of cisplatin-induced apoptosis and downregulation of the *p21* gene, respectively. Likewise, it was reported that UCA1 overexpression increased cisplatin-resistant bladder cancer; it increases WNT6 expression which, in turn, regulates the WNT signaling pathway positively [119]. It was demonstrated that NEAT1 regulated cisplatin resistance in nasopharyngeal carcinoma by targeting Rsf-1 [120]. Conversely, in lung cancer, NEAT1 enhanced cisplatin sensitivity by upregulating CTR1 [121]. Research in this area will lead to identifying potential therapy targets with the purpose of eventually avoiding or reverting resistance, the principal obstacle of treatment success.

Thankfully, lnRNAs are an en vogue topic; thus, in addition to the expression profiles, more biological and clinical studies involving signaling pathways, biological mechanisms, stages, and subtypes are surely in the pipeline, and our understanding of their role in drug resistance will only broaden in the foreseeable future.

## Figures and Tables

**Table 1:** Long non-coding RNAs associated with resistance to systemic treatments.

LncRNA	Predictive Target	Pathway Regulated	Reference
**Aromatase Inhibitors**			
**↑MIR205HG**	ERα	PI3K/AKT	[18]
**Tamoxifen**			
↑RP11.259N19.1		PI3K/AKT, focal adhesions and WNT signaling	[19]
↑KB.1460A1.5			
↑PP14571			
↓PINK1.AS			
↓KLF3.AS1			
↓LINC00339			
↓LINC00472			
↓RP11.351I21.11			
↓PKD1P6.NPIPP1			
↓PDCD4.AS1			
↓KLF3.AS1			
↓PP14571			
↓RP11.69E11.4			
↑UCA1		WNT/β-catenin signaling	[20]
↑UCA1		AKT/mTOR	[21]
↑UCA1	miR-18a → HIF1α	HIF1α signaling	[22]
↑CCAT2		Apoptosis/cell proliferation	[23]
↑ROR	miR-205-5p → *ZEB1*, *ZEB2*	Epithelial mesenquimal Transition/autophagy	[24]
↑HOTAIR	ER		[25]
↑BCAR4		Cell proliferation	[26]
**Trastuzumab**			
↑ATB	miR-200c → ZEB1, ZNF-217	Epithelial mesenquimal transition	[27]
↓GAS5	miR-21 → PTEN	Cell proliferation	[28]
↑UCA1	miR-18a → YAP1	PI3K and MAPK signaling	[29]
**Paclitaxel**			
↑H19	LIK and LOXA	Apoptosis	[30]
↑MA-Linc1		Apoptosis and cell cycle	[31]
↑HIF1A-AS2		Metabolism and Cell division cells	[32]
↑AK124454			
↑ROR		Epithelial mesenquimal transition	[33]
↑ST8SIA3			
↑MAPT-AS1	MAPT		[34]
**5-FU**			
↑NEAT1	miR-211 → HMGA	Epithelial mesenquimal transition	[35]
↑LINP1		Epithelial mesenquimal transition	[36]
↑ROR		Invasion capability	[33,37]
**Anthracyclines**			
↑ARA		MAPK signaling, focal adhesion, PPAR and metabolism signaling pathways	[38]
↑NONHSAT057282	ELF1 and E2F1		[39]
↑NONHSAG023333			
↑PANDA			[40]
↑H19		*H19-CUL4A-ABCB1/MDR1* axis	[41]

The arrows indicate over and underexpression.
